# Lipoprotein Levels in Early Adulthood and NAFLD in Midlife: The Coronary Artery Risk Development in Young Adults (CARDIA) Study

**DOI:** 10.1155/2022/1727711

**Published:** 2022-04-14

**Authors:** Sahil Khanna, John T. Wilkins, Hongyan Ning, Norrina B. Allen, Cora E. Lewis, J. Jeffrey Carr, Donald Lloyd-Jones, Lisa B. VanWagner

**Affiliations:** ^1^Division of Gastroenterology & Hepatology, Department of Medicine, Northwestern University Feinberg School of Medicine, Chicago, IL 60611, USA; ^2^Department of Preventive Medicine, Northwestern University Feinberg School of Medicine, Chicago, IL 60611, USA; ^3^Division of Cardiology, Department of Medicine, Northwestern University Feinberg School of Medicine, Chicago, IL 60611, USA; ^4^Department of Epidemiology, University of Alabama at Birmingham, Birmingham, AL 35294, USA; ^5^Department of Radiology, Vanderbilt University Medical Center, Nashville, TN 37232, USA

## Abstract

**Objective:**

We evaluated the association of apolipoprotein B (apoB) with low-density lipoprotein cholesterol (LDL-C), non-high-density lipoprotein cholesterol (non-HDL-C), and triglycerides (TG) in early adulthood with concordant/discordant associations and midlife NAFLD.

**Methods:**

Participants from the CARDIA study were included (*n* = 2,655; baseline mean age: 25.0, 59.1% female, and 48.6% black). NAFLD was defined as liver attenuation ≤40 Hounsfield units after excluding other causes of liver fat. Logistic regression models assessed the odds of Y25 NAFLD among tertiles of apoB, LDL-C, non-HDL-C, and TG and quartiles of the apoB/TG ratio. Discordance/concordance analyses examined the association of apoB with each lipid marker and Y25 NAFLD.

**Results:**

The Y25 NAFLD prevalence was 10%. The high-tertile TG group (OR 1.87, 95% CI, and 1.30–2.69) and the low- (OR 1.98, 95% CI, and 1.30–3.01) and middle-apoB/TG ratio groups (OR 1.78, 95% CI, and 1.17–2.72) had the greatest odds of midlife NAFLD. Using discordance/concordance analysis, the high-apoB/high-TG group had the highest odds of NAFLD (OR 1.69, 95% CI, and 1.09–2.61) followed by the low-apoB/high-TG group. The high apoB/low TG group had the lowest odds of NAFLD.

**Conclusions:**

Among the studied lipid markers in early adulthood, TG levels have the strongest and most consistent association with midlife NAFLD.

## 1. Introduction

Nonalcohol fatty liver disease (NAFLD) is the most common cause of chronic liver disease in the US with significant morbidity, mortality, and healthcare costs [1–4]. Lifestyle modifications have been the cornerstones for the management of NAFLD, and exercise has been shown to be beneficial in the prevention of NAFLD [5]. Therefore, early identification of individuals with higher risk for NAFLD may allow for prevention strategies that could potentially reduce both the clinical and economic burden of the disease.

Lipoprotein abnormalities can contribute to the pathophysiology of NAFLD as hepatic triglyceride (TG) overproduction and/or impaired secretion may contribute to the intrahepatic accumulation of hepatocyte-toxic lipids, inflammation, and eventual hepatic dysfunction [6]. For example, a defect in postprandial apolipoprotein B (apoB) secretion, a marker of the atherogenic particle number, may lead to TG accumulation in the liver in NAFLD-associated steatohepatitis [7, 8].

Cross-sectional studies demonstrate strong associations between high TG levels and NAFLD [6, 9, 10]. However, it is unknown if higher levels of TG or other lipoprotein markers in young adulthood are predictive of prevalent NAFLD in middle age. Of note, prior studies have treated lipoprotein variables as independent and unrelated, whereas in reality cholesterol, concentration and particle number are biologically linked and highly correlated. ApoB particles can have variable relative masses of TG and cholesterol; thus, the ratio between apoB concentration and cholesterol or TG may vary across individuals [11]; in several cohort studies, the rate of discordance varies from 11 to 25% [12, 13].

Discordance analysis compares the strength of associations to an endpoint of interest in subgroups with concordant levels of ApoB and TG/cholesterol to those with discordant levels (for example, high ApoB for the level of TG). Comparing the strength of association across groups allows one to examine the relative contribution of particle number vs lipid concentration for predicting an outcome of interest. With this in mind, the objective of our study was to use discordance analysis to examine the association of apoB with low-density lipoprotein cholesterol (LDL-C), non-high-density lipoprotein cholesterol (non-HDL-C), and TG in early adulthood with concordant or discordant associations with prevalent NAFLD in middle age. We hypothesized that higher TG levels in young adults will confer the highest odds of NAFLD in middle age.

## 2. Methods

### 2.1. Study Sample

The Coronary Artery Risk Development in Young Adults (CARDIA) study is a longitudinal, multicenter, biracial cohort study of 5,115 men and women from four geographically diverse cities (Birmingham, AL; Chicago, IL; Minneapolis, MN; and Oakland, CA) [14]. Participants were aged 18–30 years at baseline (1985–1986, Y0) and underwent follow-up examinations at years 2, 5, 7, 10, 15, 20, 25 (2010–2011, Y25), and 30; follow-up is ongoing. Among surviving participants, retention rates throughout follow-up examinations were 91%, 86%, 81%, 79%, 74%, 72%, 72%, and 71%, respectively [15]. Each participant provided written informed consent, and the institutional review boards from each field center approved the study annually.

**Figure 1:**
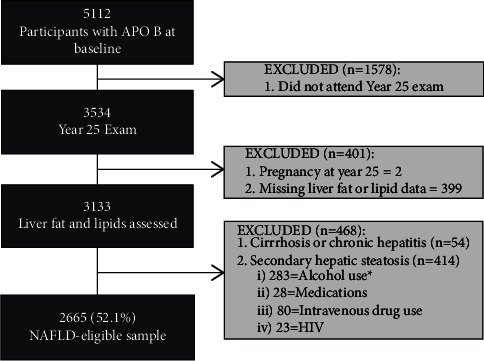
Sample population inclusion. *∗*Alcohol use was defined as >7 drinks/week for women and >14 drinks/week for men. †Medications = valproic acid, methotrexate, tamoxifen, and amiodarone.

There were 5,112 participants who had apoB levels at the Y0 examination. Participants were excluded if they did not attend the Y25 exam (*n* = 1578), they were pregnant at Y25 (*n* = 2), or their Y25 liver fat or lipid data were missing (*n* = 399). Of the remaining participants, we excluded those with self-reported cirrhosis or chronic hepatitis (*n* = 54). We also excluded participants with risk factors for potential causes of secondary hepatic steatosis (*n* = 414): alcohol consumption ≥7 drinks/week in women and ≥14 drinks/week in men (*n* = 283), medications known to cause hepatic steatosis (e.g., valproic acid, methotrexate, tamoxifen, and amiodarone, *n* = 28), intravenous drug use (*n* = 80), and self-reported HIV (*n* = 23). The remaining 2,665 participants formed the NAFLD-eligible sample population (Figure 1).

### 2.2. Measurements

Protocols for data collection across study centers and measurements have previously been described [14]. Participants self-reported demographics, medication history, alcohol consumption, and smoking status. Current smoking was defined as ≥5 cigarettes per week for ≥3 months. Physical activity was assessed using a standardized instrument; energy expenditure for moderate and vigorous activities was calculated in exercise units. Waist circumference (WC) was measured in centimeters. Body mass index (BMI) was computed by measuring weight to the nearest 0.2 kg and by measuring height to the nearest 0.5 cm [16]. Obesity was defined as BMI ≥30 kg/m^2^. Systolic and diastolic blood pressures were obtained three times at 1-minute intervals, and the second and third measures were averaged [16]. Hypertension was defined as systolic ≥140 mmHg or diastolic pressure ≥90 mmHg or the use of antihypertensive medications. Diabetes was defined as fasting plasma glucose ≥126 mg/dL or the use of an antidiabetic medication. Blood was separated into plasma and frozen at −70°C before analysis in a central laboratory. Total cholesterol (TC), high-density lipoprotein cholesterol (HDL-C), and TG were measured enzymatically, and LCL-C was estimated using the Friedewald equation [17, 18]. Non-HDL-C was calculated by subtracting HDL-C from TC. ApoB was measured by an enzyme-linked immunosorbent assay [19].

At Y25, the lower abdomen was imaged using a noncontrast computed tomography (CT) scan using GE (GE 750HD 64 and GE LightSpeed VCT 64 from Birmingham and Oakland centers, respectively; GE Healthcare) or Siemens (Sensation 64, Chicago and Minneapolis centers; Siemens Medical Solutions) multidetector CT scanners, as has been previously described [16]. NAFLD was assessed using both CT liver attenuation (LA) values ≤ 40 Hounsfield units (HU, moderate-severe NAFLD) and ≤51 HU (any NAFLD) after exclusion of secondary causes of hepatic steatosis; these thresholds have been validated previously [20–23]. LA was reported as the average of nine measurements taken on three CT slices of the right hepatic lobe. All CT scans were centrally read, and the interclass correlation coefficient between different readers on a random sample of 156 participants was 0.975 for LA, indicating high reproducibility of CT-measured LA [16].

### 2.3. Statistical Analysis

The baseline medians and tertiles for each apoB, LDL-C, non-HDL-C, TG were computed first, with correlations assessed using Spearman rank correlation, as previously described [11]. Participants were then classified into apoB, LDL-C, non-HDL-C, and TG groups based on tertiles of each measure within the entire cohort. Participants were also classified into groups based on quartiles of the ratio of apoB to TG within the entire cohort.

To study the relationship between lipid markers and to examine whether a particular lipoprotein phenotype may precede NAFLD, discordance analysis was utilized. Four mutually exclusive concordance/discordance groups were defined based on the medians of apoB and TG, LDL-C, or non-HDL-C levels: low/low (less than the median of both apoB and TG, LDL-C, or non-HDL-C), low/high (less than the median of apoB and greater than or equal to the median of TG, LDL-C, or non-HDL-C), high/low (greater than or equal to the median of apoB and less than the median of TG, LDL-C, or non-HDL-C), and high/high (greater than or equal to the medians of both apoB and TG, LDL-C, and non-HDL-C). Discordance was defined as apoB greater than or equal to the median with the other measure less than the median or vice versa.

Characteristics were compared and significance was assessed by the chi-square test for categorical variables and by analysis of variance (or Kruskal–Wallis test when appropriate) for continuous variables. Logistic regression analyses were utilized to examine the odds ratio (ORs) and 95% confidence intervals (CIs) of having moderate-severe NAFLD at Y25 among tertiles of apoB, LDL-C, non-HDL-C, and TG with the low tertile group as the referent and quartiles of the apoB/TG ratio with the high quartile group as the referent. ApoB/TG ratio quartiles were utilized as the TG distribution was more skewed and ApoB/TG quartiles allowed for a more normal distribution, similar to the distribution of other lipid markers. Logistic regression analyses were also used to model the odds of having moderate-severe NAFLD at Y25 for each set of concordance/discordance groups with the low/low group as the referent. Models were adjusted for potential confounders at Y0, including demographics (baseline age, sex, race, education, and field center) and metabolic risk factors [systolic blood pressure, antihypertensive medication use, smoking status, alcohol consumption (g/day), WC, and physical activity]. WC has been shown to be more strongly associated with NAFLD than with BMI and therefore was used instead of BMI in the final model [24]. Diabetes status was not adjusted for due to the low prevalence at Y0. Interaction terms were generated between TG tertiles, apoB/TG ratio quartiles, and apoB/TG discordance groups and race or sex for odds of NAFLD at Y25 using the fully adjusted model. Statistical significance was defined as a two-tailed *p* value <0.05.

## 3. Results

There were 2665 participants who met our inclusion criteria; at baseline, 59.1% were female and 48.6% were black, with a mean age of 25.0 ± 3.6, mean BMI of 24.5 ± 4.9; 3.5% had prevalent hypertension, and <1% had prevalent diabetes. At baseline, 22.9% were current smokers, and 11.1% had obesity. Mean LDL-C was 110.3 ± 30.8 mg/dL, non-HDL-C was 124.2 ± 33.5 mg/dL, HDL-C was 52.8 ± 12.4 mg/dL, TG was 69.7 ± 39.3 mg/dL, and TC was 177.1 ± 32.9. The mean apoB was 90.6 ± 24.1 mg/dL. The mean apoB/TG ratio was 1.54 ± 0.63.

Baseline participant characteristics stratified by tertiles of apoB concentration are presented in Table 1. Participants in the high-apoB tertile had higher WC, BMI, and prevalence of obesity than those in the low- and middle-apoB tertiles. There were no significant differences in the prevalence of smoking, alcohol use, or diabetes across the three tertiles. Systolic BP and diastolic BP were slightly higher in the high-apoB tertile, although not clinically significant.

Participant characteristics at the baseline examination according to the four apoB/TG concordance/discordance categories are presented in Table 2. Approximately 30% of participants were in each of the concordant groups (low apoB/low TG and high apoB/high TG) while approximately 18% of participants were in each of the discordant groups (low apoB/high TG and high apoB/low TG). At baseline, there were statistically significant differences in WC, BMI, and prevalence of obesity among the groups, with the highest values in the high-apoB groups. There were also statistically significant although not clinically significant differences in systolic and diastolic BP across concordance/discordance groups.

At the Y25 follow-up examination (Table 3), WC, BMI, and percentage of obesity increased across all four concordance/discordance groups (defined by baseline lipid values). Values for each of these domains continued to be higher in the high-apoB groups and were highest in the high-apoB/high TG group. There were statistically significant differences in the percentage of current smokers and alcohol drinkers across the four groups, with the most users in the higher TG groups. There were significant differences in proportion of participants with hypertension and diabetes at Y25, with the highest of each in the high-apoB/high-TG groups.

**Figure 2:**
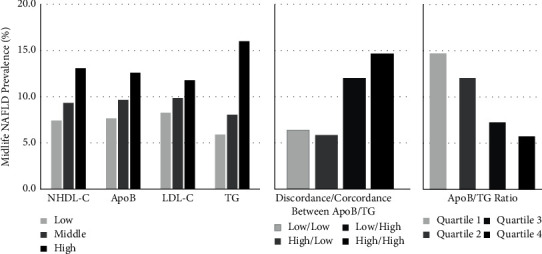
Midlife NAFLD Unadjusted Prevalence Stratified by NHDL-C, ApoB, or LDL-C tertiles, Discordance/Concordance Groups, and ApoB/TG Ratio Quartiles in Young Adulthood. Median NHDL-C: 121.0 mg/dL. Tertile ranges are low (36.0 to 107.0 mg/dL), middle (108.0 to 136.0 mg/dL), and high (137.0 to 288.0 mg/dL). Median ApoB: 88.0 mg/dL. Tertile ranges are low (22.0 to 78.0 mg/dL), middle (79.0 to 98.0 mg/dL), and high (99.0 to 292.0 mg/dL). Median LDL-C: 107.0 mg/dL. Tertile ranges are low (21 to 95 mg/dL), middle (96 to 120 mg/dL), and high (121 to 260 mg/dL). Median TG: 60.0 mg/dL. Tertile ranges are low (16 to 49 mg/dL), middle (50 to 72 mg/dL), and high (73 to 374 mg/dL).

The prevalence of moderate-severe NAFLD (herein NAFLD) at Y25 was 10% when assessed using CT LA values ≤ 40 HU. There was a direct association between each set of non-HDL-C, apoB, LDL-C, and TG tertiles in young adulthood and the prevalence of NAFLD in midlife (Figure 2). There was an inverse association between the ApoB/TG ratio in young adulthood and the prevalence of NAFLD in midlife (Figure 2), with the lowest apoB/TG ratio having the highest prevalence of NAFLD. The ORs and 95% CIs of midlife NAFLD in relation to apoB, LDL-C, non-HDL-C, and TG tertiles are shown in Table 4. The multivariable-adjusted model showed that there was difference in odds of prevalent NAFLD at Y25 in the high tertile of the TG group (OR 1.87, 95% CI, and 1.30–2.69) and the low (OR 1.98, 95% CI, and 1.30–3.01) and middle (OR 1.78, 95% CI, and 1.17–2.72) quartiles of the apoB/TG ratio groups. There was a nonlinear trend of the apoB/TG ratio, and thus, we observed the relationship between the LnApoB/TG ratio and NAFLD, which was an inverse relationship (OR 0.34, 95% CI, and 0.18–0.63).

The ORs and 95% CIs of having NAFLD at Y25 for the four concordance/discordance groups of each set of apoB and LDL-C, non-HDL-C or TG groups are also shown in Table 4. None of the apoB/LDL-C or apoB/non-HDL-C groups had a significantly higher risk of prevalent midlife NAFLD when adjusted for potential covariates. In the multivariable-adjusted model, the high-apoB/high-TG group had the highest odds of midlife NAFLD (OR 1.69, 95% CI, and 1.09–2.61) followed by the low-apoB/high-TG group. The discordant high-apoB/low-TG group had the lowest odds of midlife NAFLD. Thus, when TG was high and apoB was low or high, the odds of NAFLD were higher than that in the concordant group with high apoB/high LDL-C.

There was no significant interaction by race or sex between TG tertiles, apoB/TG ratio quartiles, or apoB/TG discordance and Y25 NAFLD status in the fully adjusted models. Therefore, race and/or sex stratified analyses are not presented. There was no substantial change in magnitude or direction of associations when fully adjusted models were additionally adjusted for cumulative exposure to lipid-lowering therapy during the 25 year follow up. Magnitude and direction of associations were also similar when the multivariable model was adjusted for Y0 BMI rather than for WC (data not shown).

## 4. Discussion

Of all lipid measures, the TG level in young adulthood had the strongest association with prevalent NAFLD over 25-years of follow up in this cohort study. First, we observed that the odds of NAFLD in midlife were highest among adults with the highest TG levels in early adulthood. Second, among concordant and discordant groups, the odds of midlife NAFLD were significantly and consistently higher when TG was greater than the median and not significantly higher than the referent when TG was below the median. Third, we demonstrated a graded and consistent inverse relationship between the apoB/TG ratio in young adulthood and prevalent NAFLD in midlife. These data suggest that the risk for midlife NAFLD is most strongly influenced by TG levels. Therefore, measuring TG levels in young adulthood may help predict midlife NAFLD better than levels of apoB, LDL-C, and non-HDL-C.

The molecular mechanisms governing hepatic lipid homeostasis include uptake of circulating lipids, de novo lipogenesis, fatty acid oxidation, and lipid export [25]. A disturbance in one or more of these pathways may precipitate hepatic fat accumulation and subsequent development of NAFLD. Fatty acid chains are hydrophobic and thus must be packaged into water-soluble very low–density lipoprotein (VLDL) particles prior to lipid export. VLDL particles are formed in the endoplasmic reticulum of hepatocytes, where one apoB molecule is combined with triglycerides, and cholesterol [26]. Endoplasmic reticulum (ER) stress has been proposed to play a critical role in both the development of hepatic steatosis and the progression to nonalcoholic steatohepatitis [27, 28]. In vivo and in vitro studies have demonstrated that prolonged exposure to fatty acids induces ER stress and post-translational degradation of apoB, subsequently leading to decreased apoB secretion [29, 30]. Thus, based on our findings and previous studies, we hypothesize that increased TG may lead to hepatic fat accumulation and subsequent NAFLD by inducing ER stress, decreasing apoB secretion, and therefore decreasing hepatic lipid export, leading to hepatic fat accumulation.

The odds of midlife NAFLD were also significantly higher when apoB was lower than the median and TG was greater than the median, suggesting that low apoB levels may be a precipitating factor in NAFLD or subclinical NAFLD. When TG levels are high yet apoB levels are low (i.e. low apoB/high TG ratio), VLDL assembly and secretion are impaired, leading to excessive lipid accumulation in the liver [26]. The relationship between NAFLD and VLDL kinetics remains unclear; however, VLDL secretion has been noted to increase in patients with NAFLD [25]. Increased intrahepatic fat content has been associated with an increased VLDL-TG secretion rate [31–34]. However, the increased VLDL-TG secretion rate plateaus when intrahepatic fat content exceeds 10%, surpassing the compensatory capacity to prevent increased hepatic fat accumulation [32]. Despite increases in VLDL-TG secretion, VLDL-apoB secretion remains largely unchanged, suggesting that individuals with NAFLD do not secrete additional particles but rather secrete larger, more TG-rich VLDL particles [25, 32]. However, the diameter of sinusoidal endothelial pores may limit secretion of very large VLDL particles. Thus, failure to compensate for limitations on VLDL particle size with an increased number of secreted VLDL particles may lead to lipid accumulation and NAFLD [35]. Low apoB or suboptimal response of apoB production may be involved in an underlying remodeling process in lipid homeostasis that proceeds to NAFLD, as suggested by the higher odds for NAFLD in the low-apoB/high-TG group.

Several cross sectional studies demonstrated that among lipid markers measured in midlife (mean ages 60.5 ± 9.6, 69.4 ± 12.8 years, and 55 ± 3, respectively), NAFLD was most associated with higher fasting serum TG levels and lower HDL-C [6, 9, 10]. Participants with elevated TG have as high as five-fold higher odds of exhibiting NAFLD than those with high total cholesterol. Our study adds to this growing body of literature by demonstrating a similar association, but taking it a step further by suggesting that measurement of TG may help identify young adults (mean age: 25 years) at risk for prevalent NAFLD in middle age (mean age: 50 years). Notably, participants in both the high TG (73 to 374 mg/dL) and middle TG tertiles (50 to 72 mg/dL) had increased odds of midlife NAFLD compared to those in the lowest TG tertile (16 to 49 mg/dL). Clinically, a substantial proportion of these participants would not traditionally be considered at “metabolic risk” based on the TG level alone. Thus, earlier identification of individuals at risk for NAFLD may allow for possible prevention and earlier intervention before adverse levels of manifesting traditional risk factors.

The primary strength of our study is the quality of the CARDIA dataset, which features a large, well-characterized biracial population cohort that has been followed longitudinally. In addition, the study population includes a moderate-to-severe NAFLD prevalence that is consistent with prior published population estimates [36]. Data analysis was also performed using a CT liver attenuation cutoff of ≤40 HU, which is specific for hepatic steatosis and has been found to identify moderate-severe steatosis compared to the gold standard of liver biopsy [20, 21].

This study has several limitations. First, these data are observational; thus, causality cannot be clearly established. Second, liver attenuation was not measured at baseline; thus, it is possible that hepatic fat was present at baseline in some participants, although we think this is unlikely because BMI was largely normal. Third, the diagnosis of NAFLD was made by CT scanning and self-reported exclusion of other causes of chronic liver disease. While CT can show features of fatty liver, liver biopsy remains the gold standard for diagnosing and classifying the severity of NAFLD [37]. Therefore, some degree of endpoint misclassification could be present; however, NAFLD is orders of magnitude more prevalent than other forms of liver disease, so we think the effect of misclassification would be modest. Finally, we did not account for the role of change in lipid markers over time and the relative association with NAFLD; however, additional research in this area is forthcoming.

Although dyslipidemia has been a well-known risk factor for NAFLD, our findings suggest that elevated TG, even levels within the “normal” range, in early adulthood, have significant positive associations with prevalent NAFLD in midlife. Further, for those with modest TG elevations, a relatively low ApoB may predict those at increased risk as well. Thus, high and high-normal TG levels in young adulthood may identify risk populations for NAFLD and may allow for targeted and intensive prevention strategies, such as dietary interventions and weight optimization. In order to better inform targeted intervention strategies, additional research is needed to examine how changes in lipid markers through early adulthood affect risk prediction for NAFLD.

## Figures and Tables

**Table 1:** Baseline characteristics by apolipoprotein B tertiles*∗*.

	Low (*n* = 883)	Middle (*n* = 885)	High (*n* = 897)	*p* value†
Age, y	24.6 (3.8)	25.1 (3.5)	25.4 (3.5)	**<0.0001**
Women, %	544 (61.6)	528 (59.7)	502 (56.0)	**0.05**
Black race, %	421 (47.7)	430 (48.6)	444 (49.5)	0.75
Education, years	14.1 (2.2)	14.1 (2.2)	14.0 (2.2)	1.00
Waist circumference, cm	74.9 (10.0)	76.6 (10.1)	80.4 (12.0)	**<0.0001**
BMI, kg/m^2^	23.5 (4.5)	24.4 (4.6)	25.5 (5.2)	**<0.0001**
Obesity (BMI>30), %	64 (7.3)	83 (9.4)	149 (16.6)	**<.0001**
Current smoking, %	205 (23.4)	189 (21.4)	217 (24.4)	0.32
Alcohol drinker, %	489 (55.6)	508 (57.5)	508 (57.0)	0.72
Physical activity, exercise unit‡	419.2 (298.8)	403.2 (290.5)	390.6 (287.6)	0.12
Systolic BP, mmHg	108.7 (10.2)	109.5 (10.0)	111.0 (11.2)	**<0.0001**
Diastolic BP, mmHg	67.7 (9.1)	68.4 (9.2)	68.8 (9.6)	**0.05**
Hypertension, %	21 (2.4)	29 (3.3)	43 (4.8)	**0.02**
Fasting glucose, mg/dL	81.2 (8.8)	81.3 (11.1)	82.9 (12.2)	**0.001**
Diabetes§, %	2 (0.2)	3 (0.3)	5 (0.6)	0.51
Lipid-lowering medication, %	0	0	0	N/A
Lipids, mg/dL				
LDL-C	82.9 (18.1)	109.0 (17.0)	138.6 (26.1)	**<0.0001**
NHDL-C	94.0 (19.1)	122.1 (17.5)	156.2 (27.2)	**<0.0001**
HDL-C	55.5 (12.3)	53.6 (12.4)	49.5 (11.7)	**<0.0001**
Triglycerides	55.0 (26.2)	65.7 (32.7)	88.2 (47.9)	**<0.0001**
ApoB	66.3 (9.6)	88.0 (5.7)	117.2 (17.5)	**<0.0001**
Total cholesterol	149.4 (21.1)	175.7 (20.2)	205.6 (28.5)	**<0.0001**

Values are in mean (SD) or number (%). *∗*The tertile ranges are low (22 to 78 mg/dL), middle (79 to 98 mg/dL), and high (99 to 292 mg/dL). †The*p* value for the test of difference across tertiles of apolipoprotein B (apoB) was determined using the chi-square test (categorical variables), analysis of variance (continuous variables) or Kruskal–Wallis test (nonparametric comparisons). ‡Physical activity was assessed using a standard instrument; energy expenditure for moderate and vigorous activities was calculated in exercise units. §Diabetes was defined as fasting glucose ≥126 mg/dL or use of antidiabetic medication.

**Table 2:** Baseline characteristics of NAFLD-eligible participants by concordance/discordance groups based on medians of ApoB/TG levels [the CARDIA study (1985–1986)].

ApoB/TG (y0)	Concordance/Discordance groups				*p* value*∗*
	Low/Low	Low/High	High/Low	High/High	
N (%)	861 (32.3)	465 (17.4)	480 (18.0)	859 (32.2)	
Age, y	24.9 (3.7)	24.6 (3.7)	25.1 (3.6)	25.4 (3.5)	**0.003**
Women, %	554 (64.3)	256 (55.1)	309 (64.4)	455 (53.0)	**<0.0001**
Black race, %	447 (51.9)	180 (38.7)	278 (57.9)	390 (45.4)	**<0.0001**
Education, years	14.1 (2.3)	14.1 (2.1)	13.9 (2.1)	14.1 (2.3)	0.44
Waist circumference, cm	74.0 (9.1)	77.7 (11.2)	76.1 (9.5)	81.1 (12.1)	**<0.0001**
BMI, kg/m^2^	23.3 (4.3)	24.5 (5.0)	24.3 (4.5)	25.7 (5.3)	**<0.0001**
Obesity (BMI>30), %	57 (6.6)	51 (11.0)	44 (9.2)	144 (16.8)	**<0.0001**
Current smoking, %	187 (21.8)	107 (23.2)	103 (21.5)	214 (25.1)	0.32
Alcohol drinker, %	455 (53.0)	290 (62.5)	259 (54.1)	501 (58.7)	**0.003**
Physical activity, exercise unit†	412.8 (291.9)	425.4 (299.8)	387.0 (291.1)	393.9 (289.1)	0.12
Systolic BP, mmHg	108.0 (9.9)	110.4 (10.2)	108.9 (10.6)	111.6 (11.0)	**<0.0001**
Diastolic BP, mmHg	67.3 (8.6)	68.6 (9.8)	67.8 (9.3)	69.5 (9.7)	**<0.0001**
Hypertension, %	12 (1.4)	19 (4.1)	15 (3.1)	47 (5.5)	**<0.0001**
Fasting glucose, mg/dL	80.8 (9.6)	81.9 (8.3)	80.9 (12.0)	83.3 (12.3)	**<0.0001**
Diabetes‡, %	3 (0.4)	0	3 (0.6)	4 (0.5)	0.43
Lipid-lowering medication, %	0	0	0	0	N/A
Lipids, mg/dL					
LDL-C	89.6 (19.7)	90.2 (19.7)	126.9 (23.1)	132.6 (27.5)	**<0.0001**
NHDL-C	98.2 (19.9)	107.1 (20.7)	136.3 (23.3)	152.9 (28.4)	**<0.0001**
HDL-C	57.3 (12.0)	50.7 (11.7)	55.3 (11.8)	48.1 (11.5)	**<0.0001**
Triglycerides	42.8 (9.8)	84.7 (28.9)	47.3 (8.6)	101.2 (45.6)	**<0.0001**
ApoB	71.1 (11.2)	73.4 (11.2)	103.7 (16.8)	112.2 (18.6)	**<0.0001**
Total cholesterol	155.5 (22.4)	157.8 (22.2)	191.7 (26.0)	201.0 (29.4)	**<0.0001**

Values shown are in mean (SD) or number (%). The median was 88.0 mg/dL for ApoB and 60.0 mg/dL for TG. *∗*Overall *P* values for comparison across groups were determined using analysis of variance for continuous variables, *χ*2 test for categorical variables, or Kruskal–Wallis test for nonparametric variables. †Physical activity was assessed using a standard instrument; energy expenditure for moderate and vigorous activities was calculated in exercise units. ‡Diabetes was defined as fasting glucose ≥126 mg/dL or use of antidiabetic medication.

**Table 3:** Y25 characteristics of NAFLD-eligible participants by concordance/discordance groups Based on medians of ApoB/TG levels [the CARDIA study (2010–2011)].

Y25 characteristics	Baseline ApoB/TG concordance/Discordance groups				*p* value*∗*
	Low/Low	Low/High	High/Low	High/High
N (%)	861 (32.3)	465 (17.4)	480 (18.0)	859 (32.2)	
Age, years	49.9 (3.7)	49.7 (3.8)	50.2 (3.6)	50.4 (3.6)	**0.003**
Waist circumference, cm	91.4 (15.2)	95.1 (16.7)	93.5 (14.6)	98.0 (16.0)	**<0.0001**
BMI, kg/m^2^	29.6 (7.3)	30.4 (7.6)	30.5 (6.9)	31.2 (7.0)	**<0.0001**
Obesity (BMI>30), %	341 (39.6)	208 (44.7)	219 (45.6)	434 (50.5)	**<0.0001**
Current smoking, %	124 (14.6)	68 (14.9)	52 (11.0)	143 (16.8)	**0.04**
Alcohol drinker, %	438 (51.4)	269 (58.6)	234 (49.4)	453 (53.1)	**0.03**
Physical activity, exercise unit†	336.7 (277.2)	346.9 (278.5)	316.6 (270.1)	336.6 (274.5)	0.39
Education, years	15.3 (2.7)	15.1 (2.7)	15.0 (2.4)	14.9 (2.8)	**0.01**
Systolic BP, mmHg	119.0 (16.1)	120.0 (17.6)	118.5 (14.6)	120.5 (15.6)	0.09
Diastolic BP, mmHg	74.0 (11.2)	75.4 (12.1)	74.6 (10.8)	75.6 (10.8)	**0.02**
Hypertension, %	255 (29.7)	163 (35.1)	160 (33.3)	347 (40.4)	**<0.0001**
Fasting glucose, mg/dL	95.2 (22.2)	99.6 (26.8)	97.4 (22.3)	104.7 (38.2)	**<0.0001**
Diabetes‡, %	63 (7.4)	52 (11.3)	41 (8.6)	138 (16.1)	**<0.0001**
Lipid-lowering medication, %	21 (2.7)	15 (3.6)	52 (11.8)	119 (15.5)	**<0.0001**
Lipids, mg/dL					
LDL-C	102.4 (26.6)	103.2 (30.0)	122.3 (32.4)	122.0 (35.7)	**<0.0001**
NHDL-C	120.4 (30.7)	128.8 (35.6)	140.7 (35.3)	148.5 (39.9)	**<0.0001**
HDL-C	61.6 (17.7)	56.6 (18.8)	60.4 (16.5)	53.2 (16.4)	**<0.0001**
Triglycerides	90.5 (65.9)	131.5 (98.7)	92.1 (52.3)	136.5 (102.3)	**<0.0001**
Total cholesterol	182.0 (31.0)	185.5 (35.2)	201.1 (36.9)	201.8 (39.9)	**<0.0001**
Liver attenuation, HU	57.5 (10.2)	54.7 (13.0)	57.0 (10.8)	53.4 (12.9)	**<0.0001**
Any NAFLD§	145 (16.8)	120 (25.8)	87 (18.1)	272 (31.7)	**<0.0001**
Moderate-severe NAFLD||	55 (6.4)	56 (12.0)	28 (5.8)	125 (14.6)	**<0.0001**

Values shown are in mean (SD) or number (%). The median was 88.0 mg/dL for ApoB and 60.0 mg/dL for TG. *∗*Overall *p* values for comparison across groups were determined using analysis of variance for continuous variables, *χ*2 test for categorical variables, or Kruskal–Wallis test for nonparametric variables. †Physical activity was assessed using a standard instrument; energy expenditure for moderate and vigorous activities was calculated in exercise units. ‡Diabetes was defined as fasting glucose ≥126 mg/dL or use of antidiabetic medication. §Any NAFLD was defined as LA < 51 HU after exclusion for other causes of liver fat. ||Moderate-severe NAFLD was defined as LA ≤ 40 HU after exclusion for other causes of liver fat.

**Table 4:** ApoB cholesterol levels and concordance/discordance between ApoB and cholesterol categories in relation to odds of Y25 moderate-severe NAFLD*∗*.

		OR (95% CI)	
	N	Unadjusted	Multivariable adjusted†
**ApoB tertiles**			
Low (referent)	883	1.00	1.00
Middle	885	1.29 (0.93, 1.81)	1.18 (0.83, 1.68)
High	897	1.74 (1.26, 2.39)	1.24 (0.88, 1.75)
**LDL-C tertiles**			
Low (referent)	898	1.00	1.00
Middle	869	1.21 (0.87, 1.67)	1.07 (0.76, 1.51)
High	898	1.47 (1.08, 2.02)	1.11 (0.79, 1.56)
**NHDL-C tertiles**			
Low (referent)	879	1.00	1.00
Middle	910	1.29 (0.92, 1.81)	1.12 (0.78, 1.59)
High	876	1.87 (1.36, 2.59)	1.26 (0.89, 1.79)
**TG tertiles**			
Low (referent)	894	1.00	1.00
Middle	884	1.41 (0.98, 2.05)	1.21 (0.82, 1.77)
High	887	3.06 (2.19, 4.27)	1.87 (1.30, 2.69)
**ApoB/TG ratio quartiles**			
Low	666	2.85 (1.93, 4.22)	1.98 (1.30, 3.01)
Middle	666	2.26 (1.51, 3.37)	1.78 (1.17, 2.72)
High	667	1.28 (0.83, 1.99)	1.21 (0.77, 1.91)
Highest (referent)	666	1.00	1.00
**ApoB/LDL-C**			
Low/low (referent)	1107	1.00	1.00
Low/high	219	0.84 (0.48, 1.46)	0.80 (0.45, 1.42)
High/low	238	1.25 (0.79, 1.99)	0.87 (0.52, 1.47)
High/high	1101	1.13 (0.84, 1.53)	0.94 (0.69, 1.29)
**ApoB/NHDL-C**			
Low/low (referent)	1125	1.00	1.00
Low/high	201	0.79 (0.44, 1.55)	0.60 (0.32, 1.12)
High/low	206	0.89 (0.51, 1.55)	0.82 (0.46, 1.47)
High/high	1133	1.46 (1.11, 1.92)	1.03 (0.76, 1.39)
**ApoB/TG**			
Low/low (referent)	861	1.00	1.00
Low/high	465	2.01 (1.36, 2.97)	1.48 (1.01, 2.24)
High/low	480	0.91 (0.57, 1.45)	0.86 (0.51, 1.45)
High/high	859	2.50 (1.79, 3.48)	1.69 (1.09, 2.61)

Median ApoB: 88.0 g/dl; median LDL-C: 107.0 mg/dL; median non-HDL-C: 121.0 mg/dL; median TG: 60 mg/dL. *∗*Moderate-severe NAFLD was defined as LA ≤40 HU after exclusion for other causes of liver fat. †The multivariable adjusted model was adjusted for age, sex, race, field center, educational attainment, + baseline (smoking status and alcohol (g/day), waist circumference, physical activity, systolic BP, antihypertensive medication use, and fasting blood glucose; ApoB/LDL-C was adjusted for TG, ApoB/TG was adjusted for LDL-C).

## Data Availability

The CARDIA Study has provided NHLBI Data Repository datasets for exams conducted during years 0–25, as well as for follow-up contacts for which data collection has been completed for at least five years, and for adjudicated morbid and mortal events. The National Heart, Lung, and Blood Institute distributes these data; additional information, including the procedures on how to request these data, can be found on the NHLBI website at https://biolincc.nhlbi.nih.gov/home/.

## Abbreviations

NAFLD: Nonalcoholic fatty liver disease
TG: Triglycerides
ApoB: Apolipoprotein B
LDL-C: Low-density lipoprotein cholesterol
Non-HDL-C: Non-high-density lipoprotein cholesterol
CARDIA: Coronary Artery Risk Development in Young Adults
Y0: Baseline (1985–1986) examination
Y25: Year 25 (2010–2011) examination
WC: Waist circumference
BMI: Body mass index
TC: Total cholesterol
HDL-C: High-density lipoprotein cholesterol
CT: Computed tomography
LA: Liver attenuation
HU: Hounsfield units
OR: Odds ratio
CI: Confidence interval
VLDL: Very low–density lipoprotein.

## Supplementary Materials

Supplementary Materials Supplemental Table 1. Baseline and Y25 Characteristics of Y25 Moderate-Severe NAFLD Participants*∗*. Supplemental Table 2. Y25 Cholesterol Levels in Relation to Odds of Y25 Moderate-Severe NAFLD*∗*.
