# Supplementary material for: Lipoprotein Levels in Early Adulthood and NAFLD in Midlife: The Coronary Artery Risk Development in Young Adults (CARDIA) Study
Source: J Nutr Metab. 2022 Apr 14;2022:1727711. doi: 10.1155/2022/1727711 (PMC9023214; doi:10.1155/2022/1727711)
Supplement: Supplementary Materials — Supplemental Table 1. Baseline and Y25 Characteristics of Y25 Moderate-Severe NAFLD Participants∗. Supplemental Table 2. Y25 Cholesterol Levels in Relation to Odds of Y25 Moderate-Severe NAFLD∗. [file 1727711.f1.docx]

**Supplemental Table 1. Baseline and Y25 Characteristics of Y25 Moderate-Severe NAFLD Participants***

|  | **Baseline**  **(1985-86)** | **Year 25**  **(2010-11)** |
| --- | --- | --- |
| Age, y | 25.4 (3.6) | 50.4 (3.6) |
| Education, years | 14.0 (2.3) | 15.0 (2.6) |
| Waist circumference, cm | 84.8 (12.4) | 111.6 (14.8) |
| BMI, kg/m^2^ | 26.7 (5.4) | 36.1 (7.5) |
| Obese (BMI>30), % | 59 (22.3) | 210 (79.5) |
| Current smoking, % | 68 (26.1) | 38 (14.6) |
| Alcohol drinker, % | 157 (59.7) | 135 (51.7) |
| Systolic BP, mmHg | 112.9 (10.5) | 124.5 (15.3) |
| Diastolic BP, mmHg | 70.0 (9.5) | 79.9 (10.3) |
| Hypertension, % | 16 (6.1) | 150 (56.8) |
| Fasting glucose, mg/dL | 83.8 (7.1) | 121.2 (45.8) |
| Diabetes†, % | 0 | 88 (33.5) |
| Lipid-lowering medication, % | 0 | 410 (15.4) |
| Lipids, mg/dL |  |  |
| LDL-C | 114.1 (32.2) | 109.9 (32.7) |
| NHDL-C | 131.8 (35.6) | 144.1 (39.5) |
| HDL-C | 48.7 (11.7) | 47.8 (15.5) |
| Triglycerides | 88.5 (52.1) | 186.5 (175.7) |
| Total Cholesterol | 180.5 (33.8) | 191.9 (39.6) |

Values are mean (SD) or number (%).

*NAFLD defined as LA ≤ 40 HU after exclusions for other causes of liver fat.

†Diabetes was defined as fasting glucose ≥126 mg/dl or use of antidiabetic medication.

Abbreviations: BMI, body mass index; BP, blood pressure; LDL-C, low-density lipoprotein cholesterol; NHDL-C, non–HDL-C (defined as total cholesterol minus HDL-C); HDL-C, high-density lipoprotein cholesterol; apoB, apolipoprotein B; TG/HDL

**Supplemental Table 2. Y25 Cholesterol Levels in Relation to Odds of Y25 Moderate-Severe NAFLD***

| **OR (95% CI)** | | | |
| --- | --- | --- | --- |
|  | **N** | **Unadjusted** | **Multivariable adjusted**† |
| **LDL-C tertiles** |  |  |  |
| Low (referent) | 868 | 1.00 | 1.00 |
| Middle | 880 | 0.84 (0.61, 1.16) | 0.88 (0.60, 1.28) |
| High | 883 | 0.84 (0.61, 1.15) | 0.81 (0.56, 1.18) |
| **NHDL-C tertiles** |  |  |  |
| Low (referent) | 871 | 1.00 | 1.00 |
| Middle | 896 | 1.16 (0.83, 1.63) | 1.04 (0.70, 1.54) |
| High | 888 | 1.82 (1.33, 2.50) | 1.34 (0.92, 1.95) |
| **TG tertiles** |  |  |  |
| Low (referent) | 893 | 1.00 | 1.00 |
| Middle | 868 | 2.66 (1.62, 4.36) | 1.81 (1.07, 3.07) |
| High | 894 | 9.74 (6.24, 15.19) | 4.19 (2.57, 6.85) |

*NAFLD defined as LA ≤ 40 HU after exclusions for other causes of liver fat.

†Multivariable adjusted model adjusted for age, sex, race, field center, educational attainment, + Y25 (smoking status, alcohol (g/day), waist circumference, physical activity, systolic BP, antihypertensive medication use, lipid lowering medication use, and fasting blood glucose. ApoB data not available at Y25.
